# Electrophysiological measurements of synaptic connectivity and plasticity in the longitudinal dentate gyrus network from mouse hippocampal slices

**DOI:** 10.1016/j.xpro.2022.102030

**Published:** 2023-01-11

**Authors:** Yoon Ji Kwon, Sojeong Pak, Sunggu Yang, Sungchil Yang

**Affiliations:** 1 Department of Neuroscience, City University of Hong Kong, 83 Tat Chee Avenue, Kowloon, Hong Kong SAR, China; 2 Department of Nano-bioengineering, Incheon National University, Incheon, South Korea; 3 Botamedi Brain Health and Medical Care Company Limited, 50 Stanley Street, Central, Hong Kong SAR, China

**Keywords:** Biophysics, Cell Biology, Microscopy, Neuroscience

## Abstract

Longitudinal synaptic connections between dentate gyrus (DG) granule neurons in the hippocampus have been found to be correlated with increased anxiety. Here, we present a protocol to assess synaptic connectivity and plasticity in the longitudinal DG network. We detail the steps for (1) obtaining acute mouse hippocampal slices that contain longitudinal DG-DG connections, (2) measuring excitatory postsynaptic potentials using whole-cell patch clamp recording combined with two-photon microscopy and glutamate uncaging, and (3) assessing synaptic plasticity using extracellular field recording.

For complete details on the use and execution of this protocol, please refer to Pak et al. (2022).^1^

## Highlights

• Obtain healthy hippocampal slices that contain intact DG-DG longitudinal synapses
• Measure postsynaptic membrane potential in whole-cell patched DG granule cells (DGGCs)
• Induce EPSPs in patched DGGCs using two-photon microscopy and glutamate uncaging
• Measure synaptic plasticity of DG-DG synapses using extracellular field recording

## Abstract (editor highlights)

Publisher’s note: Undertaking any experimental protocol requires adherence to local institutional guidelines for laboratory safety and ethics.

## Graphical abstract

**Figure:**
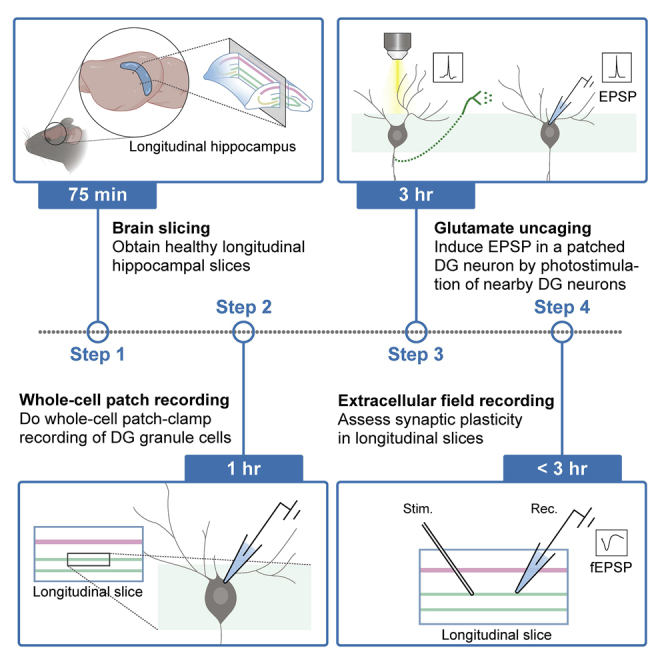

## Before you begin

All animal handling procedures were approved by the Institutional Animal Care and Use Committee of City University of Hong Kong (A-0117) and Incheon National University (INU-ANIM-2017-08).

### Before the day of the experiment

**Timing: 2 h**

**CRITICAL:** Avoid soap, detergent, ethanol, and other chemicals when washing equipment, as they may interfere with physiology.

1. Prepare the following solutions according to the materials and equipment section:
  a. 20 mL K-gluconate-based internal solution.
  b. 50 mL 1 M magnesium chloride (MgCl_2_).
  c. 50 mL 1 M calcium chloride (CaCl_2_).
  d. 2 L artificial cerebrospinal fluid (ACSF).
  e. 2 L slicing medium.

***Note:*** Prepare ACSF and slicing medium without MgCl_2_.6H_2_O and CaCl_2_.2H_2_O to prevent calcium precipitation. Add MgCl_2_.6H_2_O and CaCl_2_.2H_2_O on the day of the experiment.

2. Prepare a holding chamber.

**Figure 1:**
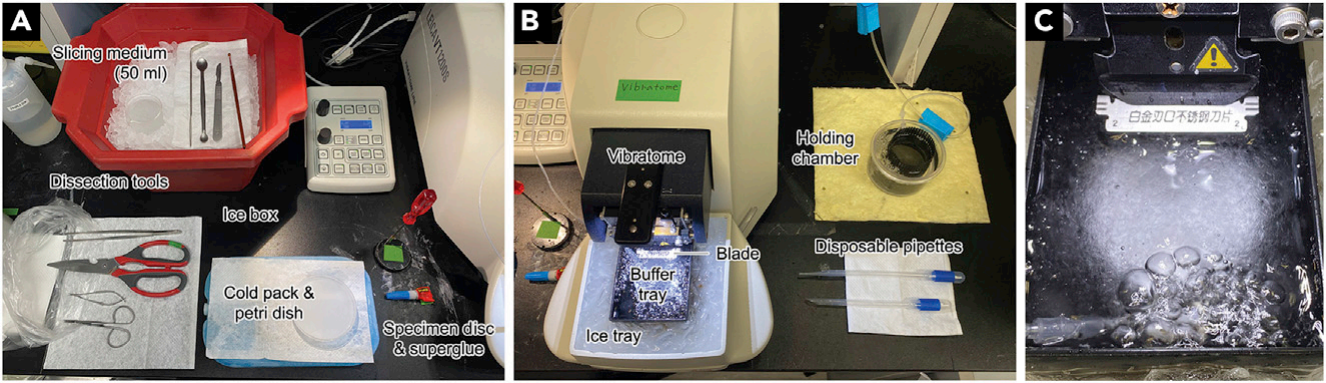
Dissection and brain-slicing tools (A) A photo example of the dissection area with ice box, cold pack, dissection tools, and specimen disc. (B) Brain slicing equipment and oxygenated holding chamber. (C) Buffer tray with an acceptable amount of frozen slicing medium crystals to start dissection.

**Figure 2:**
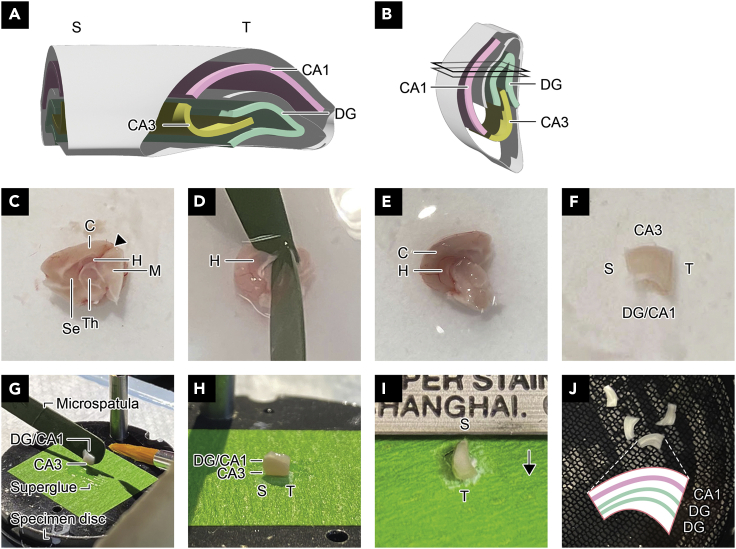
Dissection and slicing procedure to produce DG-DG longitudinal slices (A) 3D representation of the hippocampus showing a transverse cross-section at both ends as well as a longitudinal continuation of CA1 (pink), DG (green), and CA3 (yellow). (B) 3D representation of the hippocampus, oriented as in (I). Black polygons indicate the cuts made to produce slices containing DG-DG longitudinal axons. (C) A photo example of the right hemisphere of the mouse brain. The black arrowhead indicates where the blunt end of a spatula should be inserted. (D) Isolation of the hippocampus and cortex from the midbrain, thalamus and septum. The upper spatula is holding down the tissue while the bottom spatula is cutting away the midbrain, thalamus and septum. (E) Hippocampus and cortex. (F) Hippocampus with the septal and temporal ends cut out. (G) To produce longitudinal slices, the CA3 side is glued onto the specimen disc. Meanwhile, the tissue is supported by the curved end of a spatula. (H) The CA3 side is glued to the specimen disc. (I) Orientation of the hippocampal tissue in relation to the blade inside the buffer tray. The blade cuts from the septal to the temporal end (or vice versa). The black arrow indicates the direction of the blade’s movement. (J) A photo example of 400 μm-thick longitudinal slices inside a holding chamber. The inset shows a diagram of CA1 and DG layers in a longitudinal slice. **Abbreviations**: S, septal; T, temporal; DG, dentate gyrus; C, cerebral cortex; H, hippocampus; M, midbrain; Se, septum; Th, thalamus.

***Optional:*** A slice holding chamber can be crafted using a small food container (85 mm diameter), nylon stockings, plastic mesh sheet, 60 mm diameter petri dish, silicone tubing, tubing connectors, and a hot glue gun (Figure 1B). The nylon stockings, plastic mesh sheet and petri dish are assembled into an inner chamber. The inner chamber is inserted into the small food container and should be secured by hot gluing it to the bottom of the food container. It should also be surrounded by the plastic mesh sheet barrier to prevent big air bubbles from getting inside the inner chamber. The silicone tubing has small holes made with a 27G needle to allow carbogen (95% O_2_/5% CO_2_) to bubble into the outer chamber, and is placed along the outer periphery of the inner chamber. During electrophysiological recording, this inner chamber is kept submerged in ACSF or slicing medium and the brain slices are placed on top of the nylon (Figure 2J).

3. Configure stimulation protocols on [Clampex] and [Prairie View] software for whole-cell patch recording and extracellular field recording according to the manufacturers' instruction manuals.
  a. Protocol to deliver 100 ms of increasing current to a whole-cell patched neuron every 10 s for 5 min (step 36).
  b. Protocol to determine the optimal number of stimulation spots, laser duration, stimulation power and dot size for glutamate uncaging (step 47). Recommended to apply minimum power for 1 ms and increase power incrementally every 30 s.
  c. Protocol to determine the half-maximal stimulation amplitude for extracellular field recording (steps 70–74) by increasing stimulation amplitude at 30 s intervals.
  d. Protocol to deliver high-frequency stimulation (100 Hz, 1 s, 4 times at 1 s intervals) (step 77).

### On the day of the experiment

**Timing: 45 min**

4. Prepare solutions and bench:
  a. Pull a glass capillary to make micropipettes using a puller.
    ***Note:*** A glass capillary with an inner diameter of 1.65 mm is recommended. Target the tip resistance at 5–7 MΩ for DGGC whole-cell patch recording and 3–4 MΩ for extracellular field recording.
  b. Pre-chill the experiment tools (such as the dissection tools and the vibratome parts, i.e., specimen disc, buffer tray, ice tray) at −20°C for at least 15 min (Figure 1).
    **CRITICAL:** The atmospheric temperature around the dissection area should be between 18°C–20°C.
  c. Add MgCl_2_.6H_2_O and CaCl_2_.2H_2_O from its stocks to 400 mL ACSF and 400 mL slicing medium. The volume may vary depending on the length and setting of your experiment.

**Table:** 

	ACSF 400 mL	Slicing medium 400 mL
1 M MgCl_2_.6H_2_O	400 μL	2,800 μL
1 M CaCl_2_.2H_2_O	800 μL	200 μL
  d. Pre-oxygenate the slicing medium on ice with carbogen using an air stone for at least 20 min.
  e. For whole-cell patch recording, pour approx. 200 mL slicing medium into the holding chamber, and place the entire holding chamber inside a water bath set to 32°C.
  f. For extracellular field recording, pour approx. 200 mL ACSF into the holding chamber and keep it at room temperature (18°C–20°C).
    ***Note:*** Adjust the amount of solution in the holding chamber according to the size of the chamber. There should be enough solution inside to submerge the slices.
    **CRITICAL:** Oxygenate the ACSF holding chamber for at least 20 min before starting dissection. Adjust the gas pressure so that the ACSF fizzes lightly, ensuring that no big bubbles form under the nylon of the inner chamber.

5. Prepare dissection and slicing tools (Figure 1A):
  a. For example plastic bag, dissection tools (decapacitation scissors, spring scissors, forceps, scalpel), two stainless steel spatulas (one with a curved end), stainless steel spoon (1.5 cm diameter), fine brush, super glue.
  b. Cut the tip of a disposable 5 mL pipette diagonally to use it for transferring brain slices.
  c. On a flat cold pack (around 17 × 25 cm), place a petri dish with filter paper inside. The filter paper helps to prevent the tissue from slipping during dissection.
  d. Stick a piece of masking tape on the specimen disc. The brain tissue will be superglued onto the tape.
6. Chill the slicing medium for 10–15 min at −80°C until it is semi-frozen.
7. Assemble the vibratome (Figure 1B):
  a. Secure the buffer tray inside the ice tray.
  b. Fill the ice tray with ice and water while preventing any from getting inside the buffer tray.
  c. Attach the ice tray to the vibratome.
  d. Attach the blade holder and blade as in Figure 1C. Set the clearing angle at 18° (effective clearance angle is 3°). The blade is added at the last moment before beginning the slicing procedure.
  e. Lower the blade into the buffer tray.
8. Oxygenate the semi-frozen slicing medium and scrape the frozen crystals from the bottle walls with a spatula. Shake the bottle vigorously to make a slurry.
9. Fill the buffer tray with approx. 150 mL slicing medium slurry. Pour approx. 40 mL into the beaker (inside the ice box), and the remaining 10 mL into the petri dish (on top of the cold pack).
10. Oxygenate the slicing medium in the buffer tray.

***Note:*** You may need to wait until there are fewer ice crystals inside the buffer tray before proceeding with the dissection (Figure 1C), as having too many crystals may damage the brain tissue.

## Key resources table

**Table:** 

REAGENT or RESOURCE	SOURCE	IDENTIFIER
**Chemicals, peptides, and recombinant proteins**		

VetEasy Isoflurane	RWD Life Science	R510-22
Sodium chloride	Sigma-Aldrich	S7653 / CAS No. 7647-14-5
Potassium chloride	Sigma-Aldrich	P9333 / CAS No. 7447-40-7
Sodium phosphate monobasic	Sigma-Aldrich	S5011 / CAS No. 7558-80-7
Sodium bicarbonate	Sigma-Aldrich	S6297 / CAS No. 144-55-8
D-(+)-Glucose	Sigma-Aldrich	G8270 / CAS No. 50-99-7
Magnesium chloride hexahydrate	Sigma-Aldrich	M2670 / CAS No. 7791-18-6
Calcium chloride dihydrate	Sigma-Aldrich	C8106 / CAS No. 10035-04-8
Sucrose	Sigma-Aldrich	S0389 / CAS No. 57-50-1
5% CO_2_ balanced in O_2_	Linde HKO	062528G-C
Isoflurane-100 mL	RWD Sciences	R510
Potassium gluconate	Sigma-Aldrich	P1847
EGTA	Sigma-Aldrich	E0396
HEPES	Sigma-Aldrich	H7523
Adenosine 5′-triphosphate magnesium salt (ATP)	Sigma-Aldrich	A9187
Guanosine 5′-triphosphate sodium salt hydrate (GTP)	Sigma-Aldrich	G8877
Phosphocreatine di(tris)	Sigma-Aldrich	P1937
Picrotoxin	Sigma-Aldrich	P1675
MNI-caged-L-glutamate	Tocris	1490
Cadmium chloride	Sigma-Aldrich	655198
Alexa Fluor 594	Tocris	A10438

**Experimental models: Organisms/strains**		

C57BL/6J mice, 3–4 wk old, male	The Jackson Laboratory	000664

**Software and algorithms**		

Clampex (pCLAMP 10.6 Software Suite)	Molecular Devices	https://support.moleculardevices.com/s/article/Axon-pCLAMP-10-Electrophysiology-Data-Acquisition-Analysis-Software-Download-Page
ImageJ	Schneider et al.^2^	https://imagej.nih.gov/ij/

**Other**		

Faraday cage	Bruker	N/A
Fully automated vibrating blade microtome (“vibratome”)	Leica	VT1200S
Air stone	Aquarium supply store	N/A
Refrigerant Gel Bottle 400 g (“flat cold pack”)	Daiso Industries Co., Ltd.	HR-14-10
Plastic buffer tray	Leica	14048142089
Orienting specimen disc	Leica	14048142068
Super Gillette blue blades	Gillette	91169730
Decapitation scissors	Fine Science Tools	14200-21
Standard scissors	RWD Life Science	S12047-10
Spring scissors	Fine Science Tools	15003-08
Narrow pattern forceps	Fine Science Tools	11002-14
Scalpel handle	Fine Science Tools	10003-12
Carbon steel surgical blades No.10	Swann Morton Limited	0101
8/10 stainless steel double spatula, tapered	Bochem	3170
SP Scienceware™ Stainless-Steel Spoons	Bel-Art	H36729-0015
Fine brush	Artrend	11-2
Advanced Formula Super Glue Gel	Scotch	AD122
Transfer pipette, 5 mL polyethylene	Heathrow Scientific	HS206371A
Remel Plastic Petri Dishes, 85 mm	Thermo Scientific	R80150
Grade 1 filter paper, 85 mm	Whatman	1001-085
Scotch® Performance Masking Tape, 24 mm × 55 m	3 M	70006246493
Optical table	Newport	RS2000
Fixed stage microscope	Olympus	BX51WI
Optic Dodt-Gradient-Contrast System, Control and Keypad DGC_mot_	Luigs & Neumann	200-100 200 0158
Ultima In Vitro Multiphoton Microscope System	Bruker	N/A
Chameleon Vision Laser I, Laser II & Power Supply	Coherent	N/A
MRU Air Recirculator	Coherent	MRU X1
HEC-A Series Peltier-Type Chiller Thermo-con (Air-cooled)	SMC	HEC002-A
Low Profile Open Diamond Bath Imaging Chamber	Warner Instruments	RC-26GLP
Retiga R1 CCD Camera	QImaging	N/A
LUMPLFLN40XW Objective (water immersion 40× objective)	Olympus	1-U2M587
PLN10× (10× objective)	Olympus	1-U2B223
Pulse-free dispensing gear pump (perfusion input)	ISMATEC	REGLO-Z Digital
Precise peristaltic pump (perfusion output)	Longer Precision Pump	BT100-2J
Single channel temperature controller	Warner Instruments	TC-324C
Multi micromanipulator/X-Y translator/stage/microscope objective mover controller	Sutter Instrument	MPC-200
Rotary optical encoder user interface device for MPC-200	Sutter Instrument	ROE-200
Master-9 Programmable Pulse Stimulator	A.M.P.I.	Master-9
Puller	Narishige	PC-10
Concentric bipolar microelectrode (25 mm inner pole diameter)	FHC Inc	CBAPC75
4IN PTCH CLMP GL 1.5 MM (glass microcapillary tube)	World Precision Instruments	PG52151-4
4IN PTCH CLMP 1.65 MM DD (glass microcapillary tube)	World Precision Instruments	PG52165-4
1cc luer slip syringe w/o needle	Terumo	SS+01T
Masterflex Transfer Tubing, Tygon® E-Lab (E-3603), 1/16″ ID × 1/8″ OD; 50 Ft	Cole Parmer	EW-06407-71
Miniature barbed polypropylene fittings	Cole Parmer	6365-90
Pipette holder	Axon Instruments	1-HL-U
PFA-coated silver wire	A-M Systems	787000
Current clamp and voltage clamp headstage (MultiClamp 700B headstage)	Axon Instruments	CV-7B
Digitizer	Molecular Devices	The Axon™ Digidata® 1550B plus HumSilencer®
Slice anchors & kits (harps)	Warner Instruments	SHD-22CF/15 (WI64-1413)

## Materials and equipment

### Stock solution preparation

**Table:** ACSF

Reagent	Final concentration (mM)	Amount (g) per 2 L
NaCl	125.0	14.6100
KCl	2.5	0.3727
NaH_2_PO_4_	1.3	0.3119
NaHCO_3_	25.0	4.2005
D-(+)-Glucose	25.0	9.0080
MgCl_2_.6H_2_O	1.0	N/A
CaCl_2_.2H_2_O	2.0	N/A

***Note:*** Prepare with Milli-Q water. Adjust to pH 7.25–7.30 and 305 mOsm. Store at 4°C. Recommended to prepare fresh ACSF at least every week.

**Table:** Slicing medium

Reagent	Final concentration (mM)	Amount (g) per 2 L
NaCl	87.0	10.1686
KCl	2.5	0.3727
NaH_2_PO_4_	1.3	0.3119
NaHCO_3_	25.0	4.2005
D-(+)-Glucose	25.0	9.0080
Sucrose	75.0	51.345
MgCl_2_.6H_2_O	7.0	N/A
CaCl_2_.2H_2_O	0.5	N/A

***Note:*** Prepare with Milli-Q water. Store at 4°C. Recommended to prepare fresh slicing medium at least every week.

**Table:** 1 M MgCl_2_

Reagent	Amount (g) per 50 mL
MgCl_2_.6H_2_O	10.1650

***Note:*** Prepare with Milli-Q water. Store at 4°C. Recommended to prepare fresh chemical stocks at least every 2 months.

**Table:** 1 M CaCl_2_

Reagent	Amount (g) per 50 mL
CaCl_2_.2H_2_O	7.3505

***Note:*** Prepare with Milli-Q water. Store at 4°C. Recommended to prepare fresh chemical stocks at least every 2 months.

**Table:** Internal solution

Reagent	Final concentration (mM)	Amount (mg) per 20 mL
NaCl	7.0	8.182
EGTA	0.5	3.804
HEPES	10.0	47.66
K gluconate	135.0	632.3
Mg-ATP	2.0	20.28
Na_2_-GTP	0.3	3.139
Phosphocreatine di(tris)		90.68

***Note:*** Prepare with Milli-Q water. Adjust to pH 7.2–7.3 with 1 M KOH (approx. 200 μL) and 290 mOsm. Aliquot 500 μL into 1.5 mL tubes and store at −80°C. Recommended to prepare fresh internal solution every 6 months.

## Step-by-step method details

### Brain slicing

**Timing: 15 min**

**Timing: 30 s (for step 3)**

**Timing: 40 s (for step 4)**

**Timing: 6 min (for step 5)**

Recently, dentate gyrus granule cells (DGGCs) were discovered to target adjacent DGGCs^3^^,^^4^^,^^5^ via longitudinal axons that span across the dorsoventral (or septotemporal) axis of the hippocampus.^6^^,^^7^^,^^8^ Unlike transverse axons along the DG-CA3 network, these DG-DG axons are involved in anxiety-like behavior.^1^

Steps 1–7 detail the steps for obtaining acute longitudinal dentate gyrus slices in order to measure the extracellular excitatory postsynaptic potentials (fEPSPs) and examine the synaptic transmission and plasticity of longitudinal DG-DG synapses.

1. Anaesthetize a 3–4-week-old mouse by isoflurane inhalation in a closed chamber inside a fume hood.
2. When its breathing slows to around one beat per sec, decapacitate with scissors at the cervicothoracic junction to remove the spinal cord from the base of the skull.
3. Isolate the whole brain with dissection tools, being careful not to touch the hippocampus.
  a. Cut the skin from the base of the skull toward the nose using standard scissors. Peel and cut away the skin on the top and sides to completely expose the skull.
  b. Make lateral cuts on both sides of the vertebrae and cut through the jaw bones.
  c. Insert one blade of the microscissors under the skull and cut towards the eyes along the midline. At the bregma line, make two lateral cuts on the skull from the midline towards the eyes.
  d. Use forceps to open the skull from the incision and expose the whole brain.
    **CRITICAL:** Avoid applying pressure onto the brain while removing the skull.
  e. Insert the thin, flat end of the spatula near the olfactory bulb and scoop up the brain from the ventral side. Gently cut the optic and cranial nerves with the spatula to free the brain from the skull.
  f. Gently submerge the brain into the 50 mL beaker containing the slicing medium for 20 s on ice. This hardens the tissue, which facilitates slicing.
4. Dissect out the hippocampus and transfer it to the vibratome.
  a. Transfer the brain onto the cold petri dish. Use a stainless-steel spoon to transfer the brain, ensuring that only the rostroventral side of the brain comes in contact with the spoon.
  b. Cut out the cerebellum using a scalpel.
  c. Separate the left and right hemispheres with a clean top-down cut using a scalpel (Figure 2C).
  d. Insert the blunt end of one spatula into the gap between the cerebral cortex/hippocampus and the midbrain (Figure 2D). Use this to hold down the tissue but avoid pressing the spatula onto the cerebral cortex/hippocampus.
  e. Use the other spatula to cut away the midbrain, thalamus, and septum. This will expose the ‘pocket’ where the hippocampus is located.
  f. Gently isolate the hippocampus from the cerebral cortex using small, scraping motions with the blunt end (Figure 2E).
  g. Cut out the septal (medial) and temporal ends (lateral) of the hippocampus using a scalpel (Figure 2F).
    ***Note:*** Cut the septal and temporal ends at different angles to help you recognize the orientation at later steps.
  h. Spread a thin, even layer of superglue on the masking tape. Use just enough glue to cover the size of the block of the hippocampus (approx. 3 × 3 mm).
  i. Transfer the hippocampus onto the curved side of the spatula using a fine brush. Touch the brush on a piece of tissue paper to wick away any excess solution from the brain.
  j. To produce longitudinal slices, stick the CA3 (rostral) side on the glue (Figures 2G and 2H). This will orient the tissue so that the blade cuts parallel to the dorsoventral/septotemporal plane.
  k. Submerge the disc inside the buffer tray.
    ***Note:*** Orient the disc so that the tissue is sliced from the septal to the temporal end (or vice versa) rather than from CA1 to DG (or vice versa) (Figure 2I).

5. Produce hippocampal slices using the vibratome.
  a. Set the thickness to 300 μm (whole-cell patch recording) or 400 μm (extracellular field recording).
  b. 0.05 mm/s speed and 1.20 mm amplitude are recommended.
  c. Cut away the top 500–600 μm of tissue (depending on the age of the animal), and slice until the CA1 and DG are visible. When visualized under light, the CA1 is clearly visible with bare eyes.

***Note:*** The best longitudinal slices will contain one layer of CA1 and two layers of DG when observed under a light microscope (Figure 2J). One block of the hippocampus may produce 2–3 slices.

6. Using a blunted disposable pipette, gently but quickly transfer the slices into the inner chamber of the oxygenated holding chamber containing ACSF or slicing medium (Figure 2J).

**CRITICAL:** Always keep the slices submerged. Prevent the inner chamber from floating up. Remove any air bubbles from the inner chamber using a pipette, but avoid touching the slices.

***Note:*** The middle part of the chamber provides the best oxygenation for slices.

### Recover slices

**Timing: 60 min**

7. Let the brain slices rest inside the holding chamber, which is to be continuously oxygenated.
  a. For whole-cell patch recording, place the entire holding chamber inside a 32°C water bath for 30 min, then transfer it to room temperature to rest for an additional 30 min minimum.
  b. For extracellular field recording, rest the brain slices inside the holding chamber at room temperature for at least 1 h.

### Set up electrophysiology equipment

**Timing: 5 min**

8. Turn on the electronic equipment and computer software. The following should be set up on a vibration-free table.
  a. For both whole-cell patch recording and extracellular field recording:
    i. Molecular Devices Axon CNS MultiClamp 700B Microelectrode Amplifier.
    ii. Axon™ Digidata 1550B Data Acquisition System.
    iii. Molecular Devices Clampex software.
    iv. Dual-arm micromanipulator.
    v. Perfusion pumps.
    vi. Perfusion temperature controller.
  b. For whole-cell patch recording only:
    i. Ultima In Vitro Multiphoton Microscope System.
    ii. Bruker Galvanometer Driver.
    iii. Prairie Technologies Galvanometer Control.
    iv. Bruker Prairie View software.
  c. For extracellular field recording only:
    i. MASTER-9 Pulse Stimulator.
    ii. ISO-Flex Stimulus Isolator.

### Whole-cell patch clamp

**Timing: 1 h**

Steps 9–32 help achieve whole-cell patch recording of DG granule neurons (DGGCs) in longitudinal DG slices. Subsequently, focal glutamate uncaging on the dendrites of neighboring DGGCs will elicit excitatory postsynaptic potentials (EPSPs) in the patched cells (steps 33–55). EPSPs will be recorded from patched DGGCs.

***Note:*** DGGCs in slices may survive for up to 5 h after dissection.

9. Thaw an aliquot of internal solution on ice.
10. Configure the following:
  a. On one arm of the micromanipulator, mount the Current Clamp and Voltage Clamp Headstage. Then attach the silver chloride recording electrode/pipette holder (do not fit the glass micropipette yet).
  b. Adjust the angle of the Current Clamp and Voltage Clamp Headstage to 15° from the horizontal plane.
  c. Attach 1 mm diameter tubing (approx. 50 cm long) to the suction port of the pipette holder. At the other end of the tubing, attach a 1 mL syringe connected via a three-way valve.
  d. Insert a silver chloride reference electrode into the bath solution without touching the brain slice. Then ground the reference electrode.
11. Add picrotoxin to the ACSF perfusion solution (final concentration at 50 μM).
12. Perfuse the specimen stage with oxygenated ACSF maintained at 31°C–32°C by an automatic temperature controller, supplied and discharged at a steady rate (1.5 mL/min) using peristaltic pumps.

**CRITICAL:** Check frequently to make sure that the stage does not flood and that there are no excessive vibrations or fluid currents.

13. Gently transfer one brain slice to the specimen stage with a blunted disposable pipette. Place a harp on top of the slice to secure its position.

***Note:*** For glutamate uncaging, the laser path should not intersect with the pipette. Mind the orientation of the slice on the specimen stage so that the laser path can reach the molecular layer of the DG blade, but it does not contact the pipette and its path during whole-cell patch recording.

14. Acclimatize the slice on the specimen stage for an additional 30 min.
15. Visualize one slice with a 10× air objective under bright-field illumination. Check that the fluid level is stable and that there are no vibrations that could interfere with the patch process.
16. Switch to a 40× water immersion objective. Locate the dorsal blade (the DG layer closer to CA1).

***Note:*** DG granule cells can be identified by the densely packed arrangement of their soma into rows, which appear darker in color under bright-field illumination (Figure 4B).

17. Switch to a water immersion 40× objective and find a healthy cell to patch. Mark the position of the cell with masking tape on the computer monitor.

***Note:*** Healthy cells have oval-shaped soma. Unhealthy cells have shrunken, misshapen, or circular-shaped (swollen) soma with visible nuclear membranes and nucleolus. Unhealthy cells may also have a dark ‘shadow’ around the soma.

**Figure 3:**
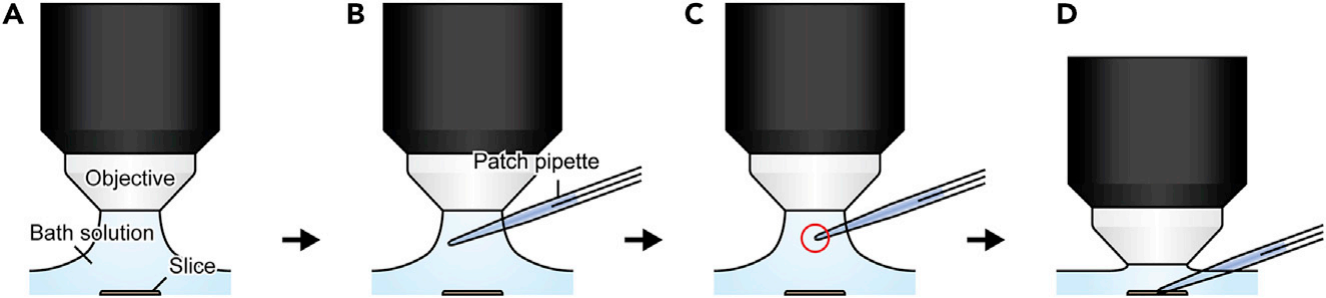
Schematic diagram of how to approach a target neuron with a patch pipette (A) The water immersion 40× objective is raised up while maintaining contact with the bath solution. (B) The patch pipette is positioned under the objective so that the shank becomes visible. (C) The tip of the pipette is found under the microscope. (D) The patch pipette and objective are lowered simultaneously until the pipette tip is just above the target neuron.
  b. With a fine control mode of the micromanipulator, position the patch pipette just below the lens until it becomes visible as a shadow under the microscope (Figure 3B).

18. Add Alexa Fluor 594 to the K-based internal solution (20–50 μM) and backfill the pulled glass micropipette with the mixed solution.
  a. Aspirate internal solution into a 1 mL syringe.
  b. Attach a 0.2 μm membrane filter to the syringe.
  c. Attach a 70 mm blunt tip microcannula to the filter.
  d. Gently tap the patch pipette to remove any air bubbles from the tip.
19. Attach the filled micropipette onto the pipette holder, making sure that the silver chloride wire touches the solution.
20. Set to voltage-clamp mode [VC] on [MultiClamp 700B commander]. Load the [Membrane Test] function on [Clampex] software and set it to [Bath] mode.
21. Apply constant positive pressure inside the pipette by injecting a small amount of air with the 1 mL syringe (approximately 0.2 mL).
22. Carefully position the patch pipette into the bath solution between the objective and the brain slice.
  a. Lift the objective until it just remains in contact with the bath solution (Figure 3A). Do not adjust the X and Y-axis positions of the stage, so you know where to approach the pipette.

**CRITICAL:** Keep the pipette tip clean by not touching it with anything but the brain slice.

23. As soon as the pipette touches the bath solution, click [Auto] under [Pipette Offset] on [MultiClamp 700B] software.
24. Check the tip resistance. The Rt in [Clampex] [Membrane Test], as well as the Resistance in [MultiClamp], should be the same.
  a. For DGGCs, target the tip resistance to be 5–7 MΩ for whole-cell patch recording and 3–4 MΩ for extracellular field recording.
25. Check the waveform of the membrane current and remove any electrical noise (see troubleshooting – problem 2) by monitoring the [Scope] window (10–20 kHz sampling rate).
26. Move the patch pipette until the tip becomes clearly visible under the microscope (Figure 3C).
  a. Check that the tip is not broken and that there are no debris or air bubbles inside.
27. Lower the pipette towards the slice while maintaining the tip in focus (Figure 3D). Use a finer control mode on the micromanipulator and the fine adjustment knob of the microscope.
28. Once the target neuron and pipette tip are visible at almost the same Z-axis position, focus on the target neuron and slowly approach the pipette towards the cell.
  a. When they come into contact, the positive pressure inside the pipette should produce a slight dent on the cell surface. The current pulse shown on the [Membrane Test] should also start to decrease.
29. *Cell attachment* – Immediately apply gentle suction by pulling out the syringe plunger or via mouth pipetting. The cell is successfully attached when the tip resistance reaches at least 1–5 GΩ.

***Note:*** Holding the membrane potential at -81 mV in [MultiClamp], which is the typical resting membrane potential of DGGCs, helps to counteract membrane hyperexcitability that may occur immediately after breaking into a patched cell. The seal should form within around 1 min.

30. Click Cp Fast: [Auto] in [MultiClamp] to correct for fast capacitance.
31. *Break-in* – Apply more potent suction to rupture the cell membrane.

***Optional:*** Use the [Zap] function in [MultiClamp] to deliver a pulse that may help with membrane rupture.

32. 1 min later, switch to current-clamp mode [IC] and measure the resting membrane potential (mV).

### Configuration of two-photon laser microscope

**Timing: 15 min**

33. Configure a two-photon laser microscope (e.g., Bruker Ultima In Vitro two-photon microscope) equipped with two lasers (e.g., Coherent Chameleon Ultra Ti: Sapphire), each of which is tuned to an excitation wavelength of 810 nm (for image acquisition) or 720 nm (for glutamate poststimulation) and modulated by an electro-optic modulator (or Pockel cell, Conotopics, M350).

***Note:*** It may take approx. 10 min for the laser to warm up after turning on the power.

***Note:*** The microscope is set up so that the epi-fluorescent and trans-fluorescent signals are captured through a 60×, 1.0 N.A. objective and a 1.4 N.A. oil-immersion condenser (Olympus). The fluorescence is split into red and green channels using dichroic mirrors and band-pass filters (ET545/30× and ET620/60 m, dichroic T570LP; ET470/40× and ET525/50 m dichroic T495LPXR). Red fluorescence (Alexa Fluor 594) signals are captured using R9110 photomultiplier tubes. Prairie View 5.4 software (Bruker) is used for image acquisition and photostimulation.

### Two-photon imaging of a patched neuron and its neurites

**Timing: 45 min**

Steps 34–43 allow high-resolution visualization of a single DG neuron that was patched in steps 9–32.

34. External light sources should be minimized in a darkened room, including any halogen light sources.
35. Switch to a 60× objective lens.
36. On [Clampex], run a pre-configured protocol to inject the threshold current (pA) once every 10 s for 5 min.
  a. The threshold current is determined as the lowest current level required to induce a single action potential.
  b. Constant threshold current (pA) is applied for 100 ms.

***Note:*** This process helps to monitor neuronal viability and promotes the fluorescence dye to spread to distal dendrites for clear visualization.

37. In the main window of [Prairie View 5.4] software:
  a. Start with 256 × 256 in [Image Resolution] field.
  b. Activate [Ch1 (Channel 1)] button in [Image Window].
  c. Click on [Live Scan] in [Scanning] field.
38. In [Power/Gain] tab, increase the power of the [Imaging] laser by clicking the right arrowhead.

**CRITICAL:** Increase the power one by one to prevent photodamaging the brain slice with the high laser intensity.

39. In [Power/Gain] tab, adjust the PMT intensity until an appropriate amount of brightness is visible in [Image Window].
40. Define a region of interest around the cell body of the patched neuron and its neurites.
  a. Set [Image resolution] at 512 × 512 with [Average Every] 4 Frames in [Scanning] field or 1024 × 1024 with 2 Frames.

***Optional:*** Increasing [Dwell Time Per Pixel (μs)] may give a clearer image.

**Figure 4:**
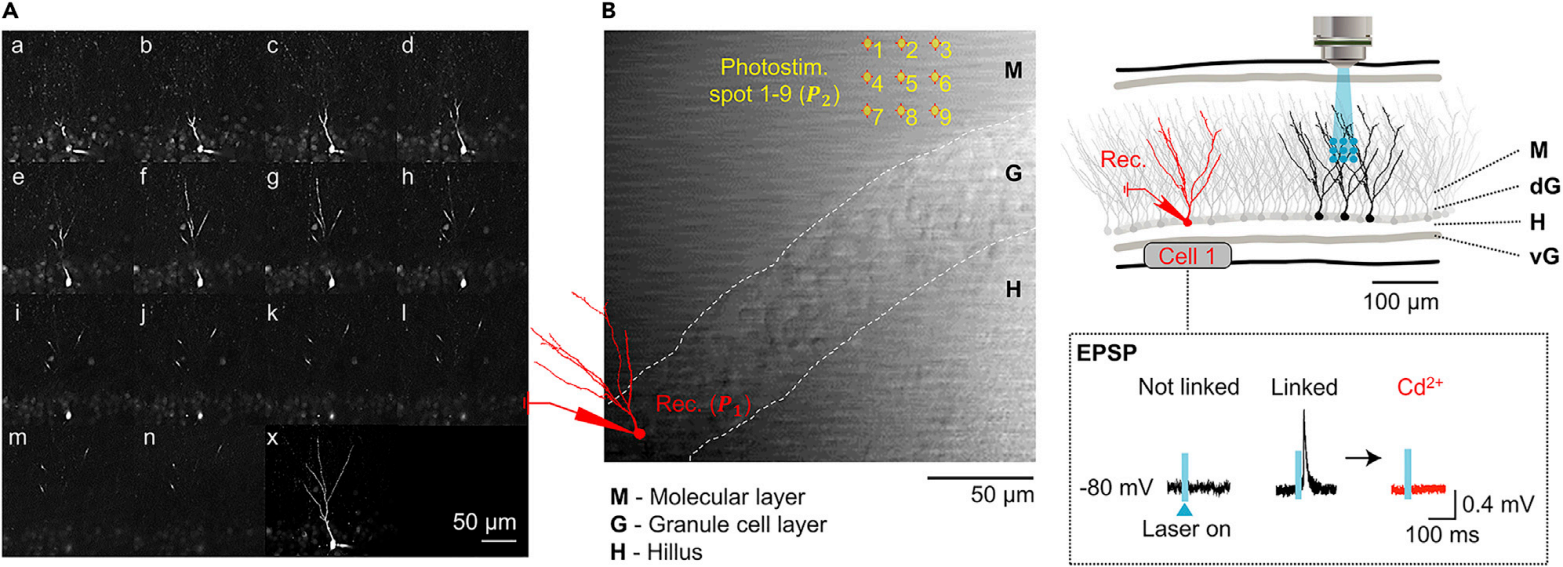
Neuronal morphology and glutamate uncaging using two-photon microscopy combined with whole-cell patch recording (A) Serial images from the top (a) to the bottom (n) of a patched neuron (Z-step = 2 μm) and a reconstructed image of neuronal morphology from multiple stacks on a spatial scale (x) showing a cell body and dendrites. (B) Example configuration of a longitudinal hippocampal slice with dentate gyrus layers visualized with Dodt gradient contrast. Circled crosshairs indicate foci where two-photon laser stimulations were applied sequentially from number 1–9. Inset shows example EPSPs of a patched cell (Cell 1) after glutamate activation of longitudinal DGGCs that have synaptic connections to the patched cell (Linked), or don’t have synaptic connections to it (Not linked). The EPSP disappears after cadmium chloride, a calcium channel blocker, treatment (Cd^2+^). Figure reprinted with permission from.^1^**Abbreviations**: Rec., recording; Photostim., Photostimulation; M, molecular layer; d(v)G, dorsal (ventral) blade of dentate gyrus granule cell layer; H, hilus; $P_{1}$ and $P_{2}$, position 1 and 2 in xyz-space; EPSP, excitatory postsynaptic potential; DGGCs, dentate gyrus granule cells.
  b. Bring the focus to the bottom of the neuron, then click the  button to set the [Stop Position (μm)] (Figure 4A.n).
  c. Stop [Live Scan] and click [Start Z-Series].

***Note:*** [Step Size (μm)] of 1–2 μm is recommended for good-quality stacked images.

41. To obtain serial Z-stack images of the patched neuron and its neurites, configure settings in the [Z-Series] tab as below:
  a. Bring the microscope focus to the very top of the neuron (Figure 4A.a), then click the  button to set the [Start Position (μm)].

42. Place the cell body in the middle of the [Image Window] and record its position $P_{1}=\left(x_{1},y_{1},z_{1}\right)$ under [Stage Control], so that the patched neuron may be located again later. To maintain a stable whole-cell configuration, the pipette position may need to be readjusted at least every 5 min to stay close to the cell body (Figure 4B).
43. Z-stack images are automatically saved in a folder defined by the user. Alternatively, the brightness or contrast can be adjusted for individual images and later use [ImageJ] software to create a stack with specified images.

### Glutamate uncaging at presynaptic neurons

**Timing: 2 h**

Steps 44–52 describe the procedure for evoking an EPSP in the patched DG neuron by photostimulation of another DG neuron(s) located in the same DG layer (ventral or dorsal). Steps 53 and 54 repeat the procedure with cadmium chloride (CdCl_2_) to confirm that the EPSP was evoked by the vesicular release of neurotransmitters from the photostimulated DG neuron.

44. Freshly prepare 500 μM MNI-caged-L-glutamate in ACSF solution and perfuse it into the recording chamber. Wait at least 3 min for the solution to diffuse entirely throughout the recording chamber (for a perfusion speed of 1.5 mL/min).

**CRITICAL:** Maintain a stable whole-cell configuration during the whole experiment.

45. Visualize the unstained brain slice without a halogen light source using Dodt Gradient Contrast (DGC), which improves the contrast of images.
  a. Deactivate [Ch1 (Channel 1)] in [Image Window] and activate [DODT] button.
  b. In [Misc] tab, change [Mirror] to [In] in [Dodt Detector] field.
  c. Click [Live Scan] in [Scanning] field.
46. Find a target area to apply photostimulation (glutamate uncaging), which should contain neurons that are highly likely to have synaptic connections with the patched neuron.
  a. Target the molecular layer (ML) hundreds of microns away from a patched neuron.
  b. Be careful not to shoot the laser on the patch pipette.
  c. Refer to the imaging to make sure that the target area excludes any dendrites extending from the patched neuron.

***Note:*** A hippocampal longitudinal slice contains two distinct layers of dentate gyrus granule cells (GC), which are the dorsal and ventral blades of granule cell layers.^3^ Therefore, target the molecular layer that is continued from the granule cell layer of the patched granule neuron. For example, do not apply photostimulation on the molecular layer that extends from the ventral blade of GC layer, if you have patched a neuron in the dorsal blade of GC layer (Figure 4B).

47. Open [Mark Points] from [Image Window] and configure the settings as follows:
  a. Be sure to optimize the laser duration, power, number of stimulation spots, and dot size beforehand to ensure that they can elicit an action potential in a neuron.
    i. Start by applying the minimum power (10 mW) for 1 ms laser duration and increase incrementally.
    ii. Rest the slices for 30 s between stimulations to avoid hyperexcitation and to give time for presynaptic neurons to recover neurotransmitter vesicles.
  b. 3 × 3 grid of point stimulation (5 ms laser duration, 10 μm spot spacing, and various interpoint delays, > 0.12 ms) at an average power of 42 mW (ranging from 7.5 to 78.08 mW) was used previously^3^; the spot size ranged from a diffraction-limited spot of 0.2–1 mm (with laser intensity and objective dependency).
  c. Make sure to set [First Repetition] under [Wait for Trigger] as well as [Start with external trigger (Trig 1)] under [Trigger Selection] for the Mark Points to hold until you run the protocol under [IC] mode in [Clampex] software that records excitatory postsynaptic potentials (EPSPs).
  d. Stop [Live Scan].
48. Click [Run Mark Points].
49. On Clampex, click [Record] and visualize the EPSP.
50. Rest the slices for 30 s between stimulations.
51. If an EPSP was evoked, place the stimulation point grid in the middle of [Image Window] and record the position $P_{2}=\left(x_{2},y_{2},z_{2}\right)$ under [Stage Control].
52. Calculate the distance between the patched neuron and the glutamate uncaging spot (the spot where EPSPs are evoked) (Figure 4B). The approximate distance between two points $P_{1}=\left(x_{1},y_{1},z_{1}\right)$ and $P_{2}=\left(x_{2},y_{2},z_{2}\right)$ in a three-dimensional space is calculated by the following generalized distance formula:

$${d(P}_{1},P_{2})=\sqrt{{\left(x_{2}-x_{1}\right)}^{2}+{\left(y_{2}-y_{1}\right)}^{2}+{\left(z_{2}-z_{1}\right)}^{2}}$$

53. To confirm that the EPSP was evoked from presynaptic vesicular release, add 0.1 mM cadmium chloride (CdCl_2_) to the perfusate and wait a few minutes for it to diffuse entirely throughout the recording chamber.
54. Repeat steps 45–50 at the position $P_{2}$ and record EPSPs under the effect of CdCl_2_.

***Note:*** CdCl_2_ blocks chemical synaptic transmissions at presynaptic terminals. If the photostimulated neurons (presynaptic neurons excited by uncaged glutamate) have synaptic connections to the patched neuron (postsynaptic neuron), the peak amplitude of EPSPs of the patched neurons should decrease or disappear after the application of CdCl_2_. As the effects of CdCl_2_ are reversible, recovery of EPSPs can be observed if steps 45–50 are again repeated without CdCl_2_.

55. After the experiment, analyze the peak amplitudes of all EPSPs offline using [Clampfit] software.

### Extracellular field recording

**Timing: 45 min**

Extracellular field recording using longitudinal DG slices allows assessment of the long-term synaptic plasticity of the DG-DG network. Steps 56–69 help to prepare brain slices for extracellular field recording.

56. After producing 400 μm-thick brain slices, set up the electrophysiology equipment as follows:
  a. On one arm of the micromanipulator, secure the concentric bipolar microelectrode (stimulating electrode) that is connected to the electrical stimulus isolator.
  b. On the other arm, mount the Current Clamp and Voltage Clamp Headstage (recording electrode headstage, angled at 20°). Backfill the pulled capillary pipette (recording electrode) with ACSF and mount the glass micropipette to the Headstage.
57. Add picrotoxin to the ACSF perfusion solution (final concentration at 50 μM).
58. Perfuse the specimen stage with oxygenated ACSF maintained at 31°C–32°C by an automatic temperature controller, supplied and discharged at a steady rate (1.5 mL/min) using peristaltic pumps.

**CRITICAL:** Check frequently to make sure that the stage does not flood and that there are no excessive vibrations or fluid currents.

59. Gently transfer one brain slice to the specimen stage with a blunted disposable pipette. Place a harp on top of the slice to secure its position.
60. Acclimatize the slice on the specimen stage for an additional 30 min.
61. Locate the dorsal DG blade under a 10× objective.
62. Load the [Membrane Test] function on [Clampex software].
63. Record the tip resistance as you lower the recording electrode with the micromanipulator until it touches the solution.
  a. Replace the glass tip if the resistance is out of the desired range (3–4 MΩ).
64. Visualize the signals on the Scope window at a 10–20 kHz sampling rate to check for electrical noise.
  a. Noise range within 0.2 mV is acceptable. If too high, refer to troubleshooting – problem 2).
65. Using the micromanipulator, lower the stimulating and recording electrodes to hover just above the slice.
66. Lower the stimulating electrode to lightly come into contact with the inner molecular layer (IML) of the dorsal blade of DG granule cell layer (Figure 5).

**Figure 5:**
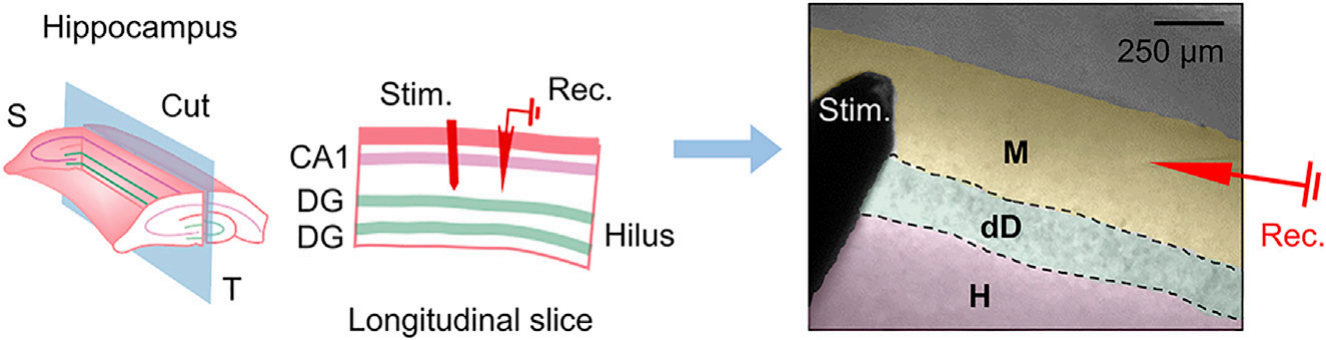
Extracellular field recording of DG-DG connections on longitudinal DG slices Illustrations of the longitudinal hippocampal slices containing DG-DG axonal connections. The recording electrode on the inner molecular layer (IML) of the dorsal DG measures field EPSPs evoked by current injection through the stimulating electrode placed on the same IML 200–400 μm away from the recording electrode. Figure reprinted with permission from.^1^**Abbreviations**: S, septal; T, temporal; Rec., recording; DG, dentate gyrus; M, molecular layer; dD, dorsal DG granule cell layer; H, hilus.
67. Lower the recording electrode to touch the same IML of the dorsal blade of DG granule cell layer 200–400 μm away from the stimulating electrode.
68. Evoke local field potentials (LFPs) with a minimum constant current at 100 ms duration and repeat at 30-s intervals.
69. In the meantime, adjust the position of the recording electrode to find the area that gives maximum amplitudes and an exemplary shape of LFPs (for reference, see Pak et al.^1^).
  a. If no responses are detected, change the location of the recording electrode, or use another brain slice.

### Input/output curve

**Timing: 20 min**

Steps 70–74 help determine the half-maximal stimulation amplitude which is the stimulation intensity that will be applied for assessing synaptic plasticity in steps 75–79.

70. Configure the digitizer, the pulse stimulator, and the electrical stimulus isolator. See the Axon Guide by Molecular Devices for information on how to set up the various equipment. Running a protocol in [Clampex] triggers the [Master-9] pulse stimulator to control the stimulus isolator to stimulate through microelectrode.
71. Set the following parameters on [Master-9 Pulse Stimulator] to evoke LFPs:
  a. Mode (M): TRIG (delivers a single pulse).
  b. Duration (D): 100 μs.
  c. Number (N): 1.
  d. Volt (V): the lowest voltage level (mV) that can evoke LFPs (e.g., ± 3 mV).
72. Find the lowest intensity in [stimulus isolator] under the [Current (I)] mode (e.g., magnification X0.1, amplitude 0.3).
73. Increase the intensity amplitude in [stimulus isolator] and record the peak amplitude of the LFPs (e.g., 0.3, 0.4, 0.5, 0.75, 1.0).

**CRITICAL:** Make sure to keep 30-sec intervals between stimulations to prevent cell damage or any changes in short-term synaptic plasticity.

74. Plot the intensity versus peak amplitude and determine the intensity that gives half of the maximum LFP amplitude. Use this intensity for all subsequent steps.

### Induce and measure long-term synaptic plasticity

**Timing: 2 h**

75. *Baseline recording* – Evoke and record LFPs every 30 s. Apply the half-maximal stimulation intensity determined above. Responses should be stable for at least 20 min before proceeding to the next step.

***Note:*** Stable synapses give constant responses over time. If unstable, change the location of the recording and/or stimulating electrode, or use the other brain slices.

76. *Conditioning* – Set the following parameters in [Master-9 Pulse Stimulator]:
  a. Mode (M): TRAIN (delivers a train of N pulses according to the programmed Delay, Duration, Interval times, and the N parameter).
  b. Duration (D): 100 μs.
  c. Interval (I): 900 μs.
  d. Number (N): 4.
77. To induce long-term potentiation (LTP), run a stimulation protocol that generates four trains of tetanus stimuli (high-frequency stimulation [HFS]; 100 Hz, 1 s, 4 times at 1- sec intervals).
78. *Post-conditioning* – Change back the [Master-9 Pulse Stimulator] settings to the parameters used in *baseline recording.* Evoke and record LFPs every 30 s for 1 h.

**CRITICAL:** Make sure that the fluid levels and the position of the two electrodes remain constant throughout the whole experiment.

79. Compare the mean peak amplitude of the last 10 min of *post-conditioning* recording to the last 10 min of *baseline* recording.

## Expected outcomes

For a whole-cell patched DG granule cell filled with Alexa Fluor 594, its cell morphology will be visible under a two-photon laser scanning microscope. Its soma, dendrites, and axons will be visible, particularly the longitudinal axons that project to neighboring DG neurons on a longitudinal DG slice. Then, EPSPs can be recorded from whole-cell patched DGGCs that are synaptically activated by a group of DGGCs excited by uncaged glutamate. On longitudinal DG slices, long-term synaptic potentiation can be induced and observed by measuring extracellular field EPSPs from the molecular layer of the DG. These expected results suggest the existence of longitudinal DG-DG connections that possess long-term synaptic plasticity.

## Limitations

It is still uncertain whether the DG-DG synaptic response in longitudinal slices solely involves DG granule neurons. Mossy cells may contribute to the recorded responses.

These methods cannot identify whether the long-term potentiation of DG-DG connections involves presynaptic mechanisms, postsynaptic mechanisms, or both.

## Troubleshooting

### Problem 1

Unhealthy brain slices (steps 32, 49, 69, 71, and 75).

### Potential solution 1

• The most important factors for obtaining healthy slices are dissecting at cold temperatures, providing sufficient oxygen, dissecting as quickly as possible, and gentle handling of tissue.
• Prepare new solutions. Check water quality, pH and osmolarity.
• Optimize the recovery period. Shorten, lengthen, or even eliminate the period in the 32°C water bath or at room temperature.
• Optimize the vibratome settings. Check that the slices have an even surface and consistent thickness. Adjust the blade speed (e.g., 1.2 mm/s) and amplitude (e.g., 1.00 mm).

### Problem 2

Excessive electrical noise (steps 25 and 64).

### Potential solution 2

• Unplug electronic devices connected to the rig one by one to find the noise source.
• Change the reference electrode. Ensure that the tip only touches the surface of the bath solution and no other surfaces of the electrophysiology set-up.
• Re-chloride the reference electrode.
• Adjust the capacitance and gain settings of the amplifier.
• Check the grounding of all components.
• For components that cannot be grounded, try shielding with aluminum foil connected to a ground box via wires and an alligator clip. The aluminum foil should not touch the table.
• Clean the pipette holder and dry it thoroughly before use.
• Check that the bath solution is not leaking from the stage.
• Salt build-up around the bath imaging chamber, or the container collecting the solution from the perfusion outlet, can contribute to noise.
• Change the positions of electronic devices nearby the rig, e.g., wifi routers.

### Problem 3

Failure to induce fEPSP in extracellular field recording (steps 72 and 73).

### Potential solution 3

• Check for the presence of fiber volley from fEPSP from the Scope window.
• If present, change the position of the electrodes, or adjust lowpass and highpass filters.
• If absent, check a different slice.
• Dead slices produce a fiber volley but no synaptic potentials.
• Prepare healthier brain slices.

### Problem 4

Unstable baseline in extracellular field recording (step 75).

### Potential solution 4

• Minimize noise.
• If cell morphology looks different from the beginning of the experiment, test a different slice.
• Minimized fluid currents in the recording chamber by placing a small piece of filter paper in the perfusion inlet port as well as the outlet port.
• Check if the slice is drifting. If so, check the slice anchor (harp), the currents from the perfusion, and the anti-vibration table.
• Check if the pipette is drifting. If so, check the micromanipulator function, replace the O-ring of the pipette holder, and give more slack to the headstage cable.

## Resource availability

### Lead contact

Further information and requests for resources and reagents should be directed to and will be fulfilled by the lead contact, Sungchil Yang (sungchil.yang@cityu.edu.hk).

### Materials availability

This study did not generate new unique reagents.

## Data Availability

This paper does not report original code.
